# Management of Asymptomatic Severe Aortic Stenosis

**DOI:** 10.2174/157340309787048103

**Published:** 2009-01

**Authors:** Robert L. Stewart, Kwan L. Chan

**Affiliations:** Division of Cardiology, University of Ottawa Heart Institute, Ottawa, Ontario, Canada

**Keywords:** Aortic valve stenosis, symptoms, management, surgery, exercise testing, brain natriuretic peptide.

## Abstract

Patients with symptomatic severe aortic stenosis (AS) benefit from aortic valve replacement surgery, but the management of patients with asymptomatic severe AS is more controversial. While cholesterol and angiotensin have been linked to AS progression, we should await the results of ongoing randomized trials before medical therapy to lower cholesterol or inhibit angiotensin can be recommended to limit disease progression. Clinical factors, echocardiographic parameters, valve morphology, exercise stress testing results, and cardiac biomarkers may be useful in identifying patients who will have early development of symptoms during follow-up and require closer monitoring. The risks associated with aortic valve replacement outweigh the benefits in the majority of patients with asymptomatic severe AS.

## INTRODUCTION

Aortic valvular stenosis (AS) develops as a result of progressive inflammation and calcification of the valve leaflets leading to restriction of leaflet excursion and left ventricular outflow obstruction. Normal aortic valve cross sectional area is 3.0 to 4.0 cm^2^ in adults and a transvalvular gradient develops when the orifice area becomes <50% of normal.

In patients with normal left ventricular systolic function, severe AS is defined as a peak AS velocity >4 m/s, a mean transaortic pressure gradient >40 mmHg, or an aortic valve area (AVA) <1 cm^2^. A valve area index <0.6 cm^2^/m^2^ is also indicative of severe AS [1].

This paper focuses on the natural history of AS, specifically looking at medical therapy that may slow the progression of the disease, and factors that can be used to risk stratify patients with asymptomatic severe AS to identify those who may benefit from surgical intervention.

## NATURAL HISTORY

The natural history of AS begins with a long asymptomatic period that is associated with minimal mortality. There is significant individual variability in the rate of progression of severity of AS. On average, peak aortic jet velocity increases by 0.32 ± 0.34 m/s per year, mean transaortic pressure gradient increases by 7 ± 7 mmHg per year, and AVA decreases by 0.12 ± 0.19 cm^2^ per year [2].

Symptoms rarely occur until the valve becomes severely stenotic. The classic symptoms of AS are exertional angina, syncope, and dyspnea (i.e. heart failure). After the onset of symptoms the average survival is only 2 to 3 years with a high risk of sudden death [3,4]. Patients with symptomatic severe AS should undergo aortic valve replacement surgery, because their survival is markedly improved following aortic valve replacement. 

Patients who are asymptomatic have a good prognosis. Overall survival in 126 patients with asymptomatic severe AS is comparable to age and sex matched controls from the general population [5]. The rate of sudden cardiac death is rare at <1% per year [2, 5, 6]. 

## PATHOLOGY

In developed countries the great majority of valvular AS is caused by calcification of a trileaflet or congenitally bicuspid or unicuspid valve [7-9]. While at one time this process was thought to be degenerative due to mechanical stress and passive calcium accumulation, it is now known to be an active one with cellular findings similar to what is seen in vascular atherosclerosis.

“Early” changes are due to subendocardial thickening secondary to chronic inflammation. This involves disruption of the basement membrane and cellular infiltration of macrophages and T-lymphocytes [10, 11]. There is also accumulation of lipids that are deposited on the leaflets and are oxidized. This ongoing process eventually leads to sclerosis and calcified deposits, particularly on the aortic side of the valve [12-15]. Calcific nodules tend to localize in the valve pockets and the base of the commissures in trileaflet valves and at the raphe and the base of the valve pockets in bicuspid valves [12-16]. The end result is thickened, stiff leaflets with limited systolic excursion.

## MEDICAL THERAPY

### Hypercholesterolemia

Lipids are central to multiple pathways in the development of fibrosis and calcification of AS. The lipid lowering and anti-inflammatory effects of hydroxymethylglutaryl-coenzyme A reductase inhibitors, or statins, and their proven disease modifying ability in atherosclerosis make them a potential agent for halting the progression of AS.

Valvular and supravalvular AS are known complications of familial hypercholesterolemia [17-20]. An association between AS progression and hypercholesterolemia has also been shown in both bicuspid and tricuspid valves [21-25]. Retrospective studies suggest that statins may slow the rate of hemodynamic progression AS [26-29]. 

Prospective studies have shown variable results. The RAAVE (Rosuvastatin Affecting Aortic Valve Endothelium) study looked at 121 patients with asymptomatic moderate to severe AS with aortic valve area of 1.0 to 1.5 cm^2^ (average peak AS velocity 3.63 m/s; average AVA 1.21 cm^2^) [30] and followed them for echocardiographic evidence of AS progression. This was an open label, prospective study in which patients with a low density lipoprotein (LDL) cholesterol >3.4 mmol/L were treated with rosuvastatin while those with an LDL <3.4 mmol/L received no lipid lowering therapy. Over the mean follow-up of 73 weeks there was reduced progression of AS in the rosuvastatin group compared to the control group (increase in AS velocity of 0.04 m/s per year in the rosuvastatin group versus 0.24 m/s per year in the control group, P=0.007; decrease in AVA of 0.05 cm^2^ per year in the rosuvastatin group versus 0.10 cm^2^ per year in the control group, P=0.041).

The effect of statin treatment on aortic valve calcification was prospectively evaluated in 61 patients with AS (mean AVA 1.16 cm^2^; range 0.7-2.0 cm^2^) who underwent baseline and one-year echocardiography and electron-beam computed tomography [31]. No difference was seen in aortic valve calcium in those treated with statin therapy versus those who were not treated over the one-year follow-up.

Currently, the only published randomized controlled trial looking at statin therapy in AS is the SALTIRE study (Scottish Aortic Stenosis and Lipid Lowering Trial, Impact on Regression) [32]. One hundred and fifty-five patients were randomized to atorvastatin 80 mg daily or placebo to test the hypothesis that intensive lipid-lowering therapy would halt the progression of AS as assessed by aortic jet velocity on Doppler echocardiography and aortic valve calcium score on computed tomography. Patients with a peak AS velocity of ≥2.5 m/s and aortic valve calcification on echocardiography were enrolled. The average peak AS velocity was 3.43 m/s, the average AVA was 1.03 cm^2^, and aortic valve calcium score was 5920 log arbitrary units. Thirty-six patients had severe AS based on a peak AS velocity ≥4.0 m/s. Patients were followed for a median of 25 months. Despite a significant change in the mean LDL cholesterol on treatment between the two groups (53% decrease in atorvastatin grouped versus no change in placebo group, P<0.001), there was no difference in measures of AS progression between the two groups (increase in peak aortic jet velocity of 0.20 m/s in both groups, P=0.95; increase in valvular calcification 22.3% per year in the atorvastatin group versus 21.7% per year in the placebo group, P=0.93) Fig. (**1**).

This study is limited by a relatively small sample size, which was not powered to assess clinical end points or small differences in valvular progression, and the large percentage of patients with severe AS, who may have too advanced disease to be amendable to medical therapy. Finally, patients with an LDL ≥4.0 mmol/L were excluded from SALTIRE, a group that might be expected to benefit from statin therapy.

Two larger clinical trials will help clarify this issue. The ASTRONOMER trial (The Aortic Stenosis Progression Observation: Measuring Effects of Rosuvastatin) has completed randomization of patients with mild to moderate AS (peak AS velocity 2.5 to 4.0 m/s) independent of valve morphology to 40 mg daily of rosuvastatin or placebo with a minimum follow-up of 3 years [33]. The SEAS study (Simvastatin and Ezetimibe in Aortic Stenosis Study) is comparing treatment with ezetimibe/simvastatin 10/40 mg daily versus placebo in patients with asymptomatic mild to moderate AS (peak AS velocity 2.5 to 4.0 m/s) with a minimum 4 years follow-up [34]. Results of both trials are expected in late 2008 or early 2009. Until the results of these large prospective studies are available, empiric statin therapy cannot be endorsed.

### Inhibition of Angiotensin

Angiotensin-converting enzyme (ACE) and angiotensin II type 1 and type 2 receptors are have been found in stenotic aortic valves [35], suggesting that the rennin-angiotensin system may play a role in the progression of the disease. ACE is found in atherosclerotic lesions and due to proinflammatory effects, contributes to the atherosclerosis.

Angiotensin inhibition has also been assessed to prevent aortic valve disease progression. Two hundred and eleven patients with asymptomatic AS with a peak AS velocity of >2.5m/s (average peak AS velocity 3.96 m/s; mean AVA 0.84 cm^2^) were retrospectively identified and the rates of hemodynamic progression of AS were compared between patients who were taking an angiotensin converting enzyme inhibitor (ACEI) versus those who were not [29]. No difference was found (increase in peak AS velocity of 0.29 m/s per year versus 0.35 m/s per year, respectively, P=0.29). This lack of treatment effect with ACEI was independent of whether or not the patients had hypertension.

The use of ACEI in preventing aortic valve calcium accumulation has also been evaluated. A retrospective analysis was performed on 123 patients who had undergone serial electron beam computed tomography for coronary calcium screening and had aortic valve calcification at baseline [36]. At a mean follow-up of 2.6 years, the no-ACEI group had a significantly higher median rate of absolute change in aortic valve calcium score than did the ACEI group (P=0.04).

While ACEI are generally used cautiously in AS due to their vasodilatory properties, they have been shown to be safe in mild to moderate AS [37] and have beneficial effects on cardiac hemodynamics [38-40], even in patients with severe AS. However, no studies have examined the effect of ACEI on clinical outcome in asymptomatic AS patients. 

In summary, there is no prospective data looking at the use of ACEI in AS. The limited retrospective data have shown contradictory results. Thus, ACEI cannot be recommended as therapy for the prevention of the progression of AS.

### Bacterial Endocarditis Prophylaxis

Recently updated American Heart Association (AHA) Guidelines no longer recommend prophylaxis of bacterial endocarditis for patients with native valvular disease including AS [41]. However, patients should continue to maintain optimal dental hygiene including regular cleanings to decrease the likelihood of sustained bacteremia.

### Summary of Medical Therapy

There is no medical therapy proven to alter the rate of progression of AS. Empiric statin therapy cannot be recommended. Trials are ongoing which should help clarify the role of statins in AS. Only limited data is available on the use of ACEI in this population. Prospective studies are needed to further assess ACEI in patients with AS.

## RISK STRATIFICATION FOR SURGICAL THERAPY

### Pitfalls of Watchful Waiting

ACC/AHA indications for aortic valve replacement in patients are as follows [1]:

- Symptomatic severe AS
- Severe AS in patients undergoing coronary artery bypass grafting, other valve surgery, or surgery on the ascending aorta
- Left ventricular systolic dysfunction (ejection fraction <50%) secondary to severe AS

Based on these guidelines, patients with asymptomatic severe AS are not recommended to undergo valve replacement surgery. However, there are concerns with this conservative approach and some advocate earlier consideration for surgical treatment [42, 43]. While the risk is low in the asymptomatic patient there continues to be a small risk of sudden cardiac death. In addition, symptoms can be subtle or hidden by a reduction in the patient’s activity level. Finally, even with a careful watch and wait approach, sudden death can occur soon after the onset of symptoms, either before the patient seeks medical attention, or while waiting for surgery.

Thus, an ideal approach to minimize the risk of adverse events would be to identify and refer patients for surgery just before the onset of symptoms. Various methods have been proposed to attempt to identify patients with asymptomatic severe AS who are at risk for adverse events. These include assessing clinical and hemodynamic predictors, valve morphology, exercise stress testing, and the use of cardiac biomarkers.

### Clinical/Hemodynamic Predictors

One hundred and twenty-three asymptomatic patients with a peak AS velocity >2.5m/s and thickened aortic leaflets with reduced excursion were followed with regular clinical assessment, echocardiography, and exercise treadmill testing for 2.5 years to determine prognostic factors [2]. Baseline peak AS velocity predicted the likelihood of aortic valve replacement. Patients with an AS velocity >4 m/s have a >50% likelihood of symptom onset or death within a 2 year period. Other independent predictors of death or aortic valve surgery in multivariate analysis were the rate of change of AS velocity over time (P<0.00001) and functional status score (P=0.02).

A large cohort of patients with asymptomatic severe AS with peak AS velocity ≥4 m/s (average peak AS velocity 4.4 m/s; mean AVA 0.9 cm^2^) were followed for 5.4 years to assess the risks and predictors of mortality in asymptomatic severe AS [44]. Only AVA (P=0.005) and left ventricular hypertrophy (P=0.04) were independent predictors of symptom onset, and development of symptoms preceded sudden death in all but 11 of 179 patients. 

In another study of patients with severe AS (peak AS velocity >4 m/s), echocardiographic assessment of AS severity (AS velocity or AVA) was not predictive of future events, nor were any other clinical or hemodynamic parameters [5].

A recent prospective study involving 133 asymptomatic patients with a peak transaortic pressure gradient ≥60 mmHg and normal left ventricular systolic function found that only a reduction in ejection fraction over time (but remaining within normal limits) was an independent predictor of symptom onset or sudden cardiac death (P=0.001) [45]. However, in individual patients the clinical utility of this predictor is limited as the change in ejection fraction is small and single measurements of ejection fraction have wide confidence limits.

### Valve Morphology

The ability of valve morphology to predict future events was assessed in 128 consecutive patients with asymptomatic severe AS (peak AS velocity >4 m/s) [5]. In this group who were followed for a mean of 24 months, only the extent of valve calcification was an independent predictor of outcome. Those with no or mild valvular calcification had significantly fewer events (death or aortic valve replacement necessitated by the onset of symptoms) than patients with moderate or severe calcification (P<0.001) Fig. (**2**). 

The combination of moderate to severe valvular calcification with a rapid increase in AS velocity was a particularly high risk combination. An increase in AS velocity of ≥0.3 m/s per year in the group of patients who had moderately or severely calcified valve leaflets predicted a 79% likelihood of surgery or death within 2 years of the observed increase. The importance of valvular calcification as a predictor of the rate of AS progression had also been shown when the calcification was determined by angiography [46].

### Exercise Stress Testing

Sixty-six patients with asymptomatic severe AS based on an AVA <1.0 cm^2^ (average peak transaortic gradient 83 mmHg; mean AVA 0.61 cm^2^) underwent treadmill exercise stress testing to determine its prognostic value [47]. The main outcome measures were development of symptoms or sudden cardiac death, and mean follow-up was 15 months. A positive stress test was defined as onset of symptoms (precordial chest pain or near syncope), significant ST depression, complex ventricular arrhythmia, or a failure of a systolic blood pressure to rise >20 mmHg during exercise. Forty-four patients had a positive test, the majority due to symptoms. A positive test was predictive of reaching an endpoint (hazard ratio 7.4). Four patients died suddenly, all of whom had a positive stress test.

One hundred and twenty-five patients with asymptomatic moderate to severe AS based on an AVA <1.4 cm^2^ (mean AVA 0.9 cm^2^) underwent exercise treadmill testing and were followed for 12 months after the stress test [48]. Endpoints were the onset of spontaneous exertional symptoms or sudden cardiovascular death. The criteria for a positive stress test were the development of limiting symptoms (chest discomfort, breathlessness, or dizziness), significant ST depression, or no increase in blood pressure at peak exercise compared to baseline. Symptoms on stress testing were an independent predictor of spontaneous symptoms during follow-up (P<0.001). An abnormal blood pressure response or ST segment depression did not improve the predictive ability of the test. No patients died in follow-up.

Exercise echocardiography has been used for risk assessment in 69 patients with asymptomatic severe AS defined by an AVA ≤1.0 cm^2 ^(average peak transaortic pressure gradient 65 mmHg; mean AVA 0.81 cm^2^) [49]. Stress testing was performed using a semi-supine bicycle. The combined end-point was the development of symptoms (angina, dyspnea, or syncope), hospital admission for heart failure, requirement for aortic valve replacement, or cardiac death. These patients were followed for 15 months. A positive stress test was defined as an onset of symptoms, significant ST depression, significant arrhythmia, or a fall or small rise (<20 mmHg) in systolic blood pressure as compared to baseline. Those who had a normal test had increased event free survival when compared to those with an abnormal test (P=0.0026). An increase in exercise induced mean transaortic pressure gradient of ≥18 mmHg (value determined from receiver-operator characteristic curve) was predictive of events (P=0.0015), and provided incremental prognostic information to resting echocardiographic and exercise electrocardiographic parameters.

Multiple studies have shown that a positive stress test has the ability to predict the onset of spontaneous symptom and thus requirement for valve replacement surgery [47-51]. The majority of abnormal or positive exercise stress test were based on symptom onset, which appear to be more predictive than other criteria [52]. Electrocardiographic ST depression has shown limited ability to predict future events. The high incidence of concomitant coronary artery disease in patients with AS may have contributed to its poor predictive ability. Variable results have been shown for the value of an abnormal blood pressure response with exercise to predict events [2, 47-51]. Differences in the definition of an abnormal blood pressure response and in the mode of stress testing (treadmill, upright bicycle, semi-supine bicycle) likely contributed to the inconsistent results. 

Contradictory to conventional opinion, carefully monitored exercise stress testing in patients with severe AS is safe. No complications were reported in the studies discussed above [47-51] which included 400 patients, most of whom had at least moderate AS. Properly monitored exercise stress testing is feasible and safe in patients with AS, provided that these patients have been stable with no overt symptoms of AS. A comprehensive history is a prerequisite for exercise testing in these patients. 

### Cardiac Biomarkers

Brain natriuretic peptide (BNP) is a neurohormone secreted by the ventricles in response to volume and pressure overload [42, 53]. BNP levels have been used to assess risk in patients with heart failure, acute coronary syndromes, and chronic severe mitral regurgitation [54-57]. BNP has also been found to be elevated in patients with AS [58-60].

BNP and its aminoterminal portion Nt-BNP are increased in patients with symptomatic AS compared to those who are asymptomatic. Natriuretic peptides progressively increase with higher New York Heart Association functional class and with lower AVA [61-66]. These finding have been consistently shown in prospective studies.

One of these studies involved 130 patients with severe AS based upon peak AS velocity >4 m/s and/or an AVA <1.0 cm^2^ (average peak AS velocity 5 m/s; mean AVA 0.64 cm^2^) [63]. Baseline BNP levels were used to assess symptom free survival in the 43 patients who were asymptomatic at the time of entry into the study. Patients were followed for mean of 377 days and those with a BNP ≥130 pg/mL or an Nt-BNP ≥80 pmol/L were significantly more likely to develop symptoms over the next 6 to 9 months (P<0.001 for both). Another study found that a BNP cut-off of ≥190 pg/mL provided the best discriminatory value for the presence of symptoms [66].

Nt-BNP but not BNP is a predictor of post-operative survival in patients who undergo aortic valve replacement [63]. In patients with severe AS and/or aortic regurgitation, Nt-BNP decreases after successful aortic valve surgery, but continues to increase in patients managed conservatively [67].

Elevated plasma BNP is clearly associated with AS severity and cardiac symptoms in patients with AS. It also has prognostic value in patients scheduled to undergo surgical treatment. However, there continue to be limitations to the utility of BNP. First, BNP values vary with age, sex [68], and body mass index [69], and can be elevated in other cardiac and non-cardiac conditions [70]. Secondly, optimal cut-offs have yet to be established as there is substantial overlap in BNP levels between AS patients with or without adverse events. Finally, while natriuretic peptides have been shown to predict symptoms, they have not yet been proven to accurately identify patients who are likely to have an adverse event prior to symptom onset.

## SUMMMARY

Many features can be used to identify patients with asymptomatic severe AS who are likely to develop early symptoms (Table **1**). 

In deciding whether to perform aortic valve replacement in asymptomatic patients with severe AS, the risk of not operating compared to the risk of surgery must be considered. Limitations associated with watchful waiting include the subjectivity of patients’ symptoms, concern about sudden death, and the risk of asymptomatic left ventricular systolic dysfunction. However, as earlier discussed, many papers have shown that the risk of sudden death is quite low at <1% per year [2, 5-6, 47-51]. So, despite the subjectivity and variability of patients’ symptoms, it remains the best indicator for the need for aortic valve replacement. Asymptomatic left ventricular systolic dysfunction is an uncommon indication for surgery. When present, aortic valve replacement typically results in restoration of systolic function.

Operative mortality for isolated aortic valve replacement is 3-4% [60, 71], higher than the risk of sudden death in asymptomatic patients. The rate of late annual mortality (>4 weeks post-operative) in patients with aortic valve replacement has been reported at 3.6%, with 24% of those deaths attributable to sudden cardiac death [72]. In addition, there is morbidity associated with a prosthesis including prosthesis dysfunction, valve thrombosis, endocarditis, embolism, and the risks associated with anticoagulation. The combined risk of these complications for an aortic prosthesis is >2% per year [73, 74]. Thus, in the great majority of patients the risks of aortic valve replacement surgery and potential long-term complications of a prosthesis are greater than the risks of careful monitoring of asymptomatic severe AS.

Assessment of patients with equivocal symptoms, such as those who are sedentary or whose history is unreliable can be challenging. In this subgroup, the markers listed in Table **1** may be useful. In particular, serious consideration should be given to performing a carefully monitored exercise test which has been shown to be safe, clarifies the symptomatic state, and provides useful hemodynamic information predictive of prognosis. 

## CONCLUSIONS

The progression of AS is highly variable. At this point there are no medical therapies that have been proven to delay the progression of AS. Aortic valve replacement is indicated for patients with symptomatic severe AS. In the great majority of patients with asymptomatic severe AS, the risk of surgery outweighs the risk of watchful waiting. There are clinical, echocardiographic and biochemical indicators which can help identify those likely to develop symptoms. A carefully monitored stress test appears safe and useful in patients with equivocal symptoms.

## Figures and Tables

**Fig. (1):**
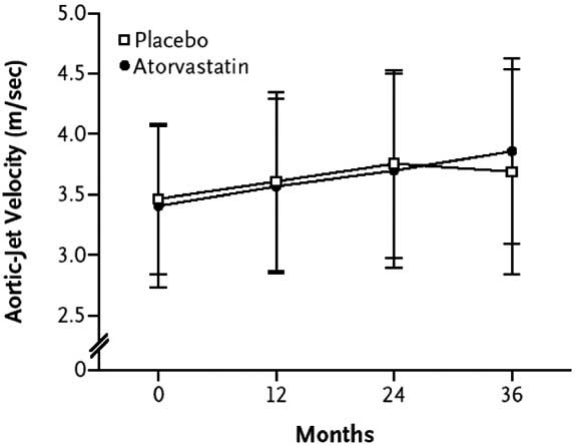
Progression of AS in patients treated with intensive atorvastatin therapy on matched placebo. [from reference 22 with permission].

**Fig. (2):**
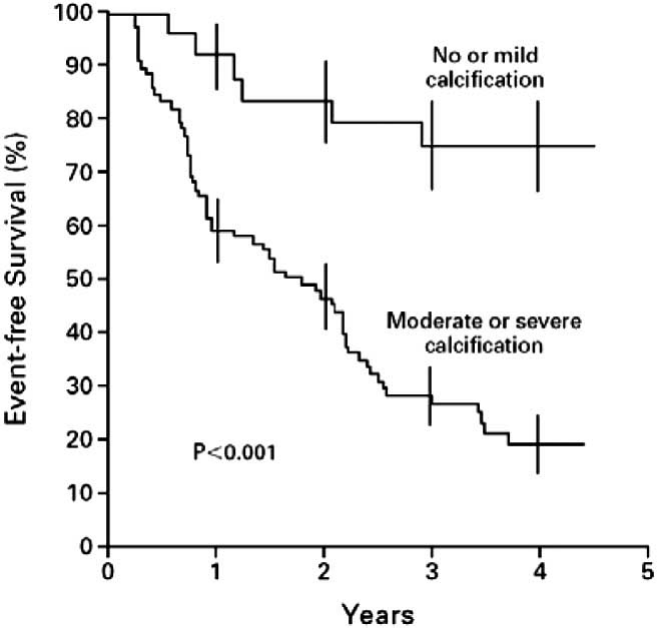
Event-free survival among patients with nor or mild aortic valve calcification compared with moderate or severe calcification. [from reference 5 with permission].

**Table 1:** Predictors of Early Development of Symptoms in Patients with Asymptomatic Severe AS

Severity of AS Increased rate of AS progression (≥0.3 m/s per year) Moderate to severe valvular calcification Abnormal exercise stress test, particularly due to symptoms Increased mean transaortic pressure gradient >18 mmHg with exercise Elevated BNP ≥130 pg/mL or Nt-BNP ≥80 pmol/L
